# Development and validation of a prediction model for failed shockwave lithotripsy of upper urinary tract calculi using computed tomography information: the S_3_HoCKwave score

**DOI:** 10.1007/s00345-020-03125-y

**Published:** 2020-02-22

**Authors:** Takashi Yoshioka, Tatsuyoshi Ikenoue, Hideaki Hashimoto, Hideo Otsuki, Tadashi Oeda, Noritaka Ishito, Ryuta Watanabe, Takashi Saika, Motoo Araki, Shunichi Fukuhara, Yosuke Yamamoto, the Okayama-Ehime S.W.L. Study Group

**Affiliations:** 1 Department of Healthcare Epidemiology, Graduate School of Medicine, Kyoto University School of Public Health, Yoshida-Konoe-machi, Sakyo-ku, Kyoto-shi, Kyoto 606-8501 Japan; 2 Center for Innovative Research for Communities and Clinical Excellence, Fukushima Medical University, 1, Hikarigaoka, Fukushima-shi, Fukushima 960-1295 Japan; 3 Kyoto University Graduate School of Medicine/Human Health Science, 53 Kawahara-cho, Sakyo-ku, Kyoto, 606-8507 Japan; 4 Department of Urology, Okayama Central Hospital, 6-3, Ishima-kita-machi, Kita-ku, Okayama-shi, Okayama 700-0017 Japan; 5 Department of Urology, Abiko Toho Hospital, 1851-1, Abiko, Abiko-shi, Chiba-ken 270-1166 Japan; 6 Department of Urology, Onomichi Municipal Hospital, 3-1170-177, Shin-Takayama, Onomichi-shi, Hiroshima 722-8503 Japan; 7 Department of Urology, Kurashiki Medical Center, 250, Bakuro-cho, Kurashiki-shi, Okayama 710-8522 Japan; 8 Department of Urology, Matsuyama Shimin Hospital, 2-6-5, Ohte-machi, Matsuyama-shi, Ehime 790-0067 Japan; 9 Department of Urology, Ehime University Graduate School of Medicine, Shizukawa, To-on-shi, Ehime 791-0295 Japan; 10 Department of Urology, Dentistry, and Pharmaceutical Sciences, Okayama University Graduate School of Medicine, 2-5-1, Shikata-cho, Kita-ku, Okayama-shi, Okayama 700-8558 Japan

**Keywords:** Urolithiasis, Extracorporeal shockwave therapy, Decision making, Decision support techniques

## Abstract

**Purpose:**

To develop and validate a new clinical prediction model that accurately predicts the failure of shockwave lithotripsy (SWL) using information obtained from non-contrast-enhanced computed tomography (NCCT).

**Methods:**

This multicentre retrospective cohort study consecutively enrolled patients diagnosed with upper urinary tract calculi by NCCT at five hospitals in Japan from January 1, 2006 to December 31, 2016. Among the candidate predictors, we selected the six most significant predictors a priori. The main outcome was SWL failure after three sessions. Model calibration was evaluated by the calibration slope and the Hosmer–Lemeshow test. Discrimination was evaluated by the receiver-operating characteristic curves and the area under the curve (AUC). A multivariable logistic regression analysis was performed; based on the estimated *β* coefficients, predictive scores were generated.

**Results:**

Of 2695 patients, 2271 were included. Patients were divided into the development cohort (1666 patients) and validation cohort (605 patients) according to geographical factors. We developed a clinical prediction model with scores ranging from 0 to 49 points. We named the prediction model the S_3_HoCKwave score based on the initials of the predictors (sex, skin-to-stone distance, size, Hounsfield units, colic, and kidney or ureter). As a result of internal validation, the optimism-corrected AUC was 0.72. In the validation cohort, the Hosmer–Lemeshow test did not show statistical significance (*P* = 0.33), and the AUC was 0.71 (95% confidence interval 0.65–0.76).

**Conclusions:**

The S_3_HoCKwave score is easy to understand, has a relatively high predictive value, and allows clinicians to make appropriate treatment selections.

**Electronic supplementary material:**

The online version of this article (10.1007/s00345-020-03125-y) contains supplementary material, which is available to authorized users.

## Introduction

Upper urinary tract calculi (i.e., kidney stones and ureteral stones) are common, with a prevalence of 5.2% during 1988–1994 [1], and increasing trends in the United States and Japan [2]. Symptoms of upper urinary tract calculi vary and are sometimes serious [3]. Upper urinary tract calculi can be complicated by sepsis, which can be fatal [4]. Therefore, early diagnosis and early treatment interventions for upper urinary tract calculi are important in clinical practice.

In Japan, 56.6% of patients diagnosed with upper urinary calculi in 2015 received some type of surgical treatment, such as shockwave lithotripsy (SWL), ureteroscopic lithotripsy (URSL), or percutaneous nephrolithotomy (PCNL) [2]. The European Association of Urology (EAU) and Japanese Urological Association (JUA) recommend the use of either SWL or URSL when a patient has a single calculi with a diameter of 20 mm or smaller [5, 6]. A recent systematic review indicated that the stone-free rate (SFR) of URSL is superior to that of SWL at 4 weeks after treatment, whereas the SFR of URSL at 3 months after treatment is equivalent to that of SWL [7]. Additionally, more complications and longer hospitalisation periods are associated with URSL than with SWL [7]. Therefore, SWL is a viable alternative treatment that may have clinical advantages over URSL for solitary calculi smaller than 20 mm.

Recently, shared decision-making (SDM) has become an important practice in urology [8], and it can be used with clinical prediction models [9]. Although there are some prediction models that predict the SWL success rate [10–14], they may be difficult to use because of their complexity. In addition, their performance has not yet been sufficiently evaluated. Therefore, our study aimed to develop and validate a novel and epidemiologically robust clinical prediction model that can provide clinically useful information regarding treatment selection to determine if SWL is appropriate for the treatment of upper urinary tract calculi.

## Patients and methods

### Research design and setting

We conducted a multicentre retrospective cohort study at five Japanese community hospitals.

### Inclusion and exclusion criteria

We consecutively included patients who were 20 years or older and were diagnosed with solitary upper urinary tract calculi by non-contrast-enhanced computed tomography (NCCT) and underwent SWL as an initial treatment from January 1, 2006 to December 31, 2016. We followed-up patients to determine outcomes for 3 months based on the recommendations of the JUA guidelines [5] and on actual practice patterns during January 1, 2006 to March 31, 2017. We excluded patients who had urinary calculi of 20 mm or larger, had lower renal calyx calculi or multiple calculi, had indwelling ureteral stents, and did not have data regarding outcomes.

### How to perform SWL

Patients were administered transrectal diclofenac and placed in the supine position to undergo treatment for upper ureter calculi or renal calculi. Treatment of mid-to-lower ureter calculi was performed with patients in the prone position. The shockwave rates were 60–90 per min. Shockwave energy was gradually increased to a tolerable level for patients, and involuntary patient movement and increased respiratory motion due to pain were prevented. The maximum number of shocks was 4000. Most SWL procedures were performed by well-trained Japanese board-certified urologists with 7 or more years of experience at each hospital.

### Main outcome measures

The primary outcome was SWL failure after three sessions, which reflected the overall SWL outcome [5, 6]. We defined treatment success as the resolution of calculus as determined by abdominal X-ray examination during the subsequent clinical outpatient visit. Clinically insignificant residual fragments, which were observed with residual stones smaller than 4 mm in diameter [15, 16], were considered to indicate successful treatment. Cases converted to URSL or PCNL were defined as SWL failure. SWL failure after one session and SWL failure after two sessions were also evaluated as a secondary outcome.

### Sample size calculation

To develop the multivariable logistic regression model, at least ten events per variable were needed [17]. At least 100 events were needed for model validation [18]. Based on the JUA and EAU guidelines, SWL failure was expected to occur in 10–30% of patients [5, 6]. We calculated that we needed 500–1500 patients for the development cohort and 330–1000 patients for the validation cohort.

### Development of a prediction model

First, we divided our cohort into two cohorts, the development cohort and the validation cohort, according to geographical factors. Second, based on previous studies, guidelines [5, 6, 11–14], and opinions from an expert panel comprising 13 urologists of our study team, the 6 most clinically significant predictors (sex, presence of colic, maximum length of calculi, localisation of calculi, skin-to-stone distance [SSD], and mean stone density) were selected a priori. Third, we converted continuous outcomes to dichotomous outcomes according to previous studies. Fourth, we used a multivariable logistic regression analysis for the development cohort and calculated the *β* coefficients of each predictor. Fifth, we rounded those *β* coefficients and multiplied by 10 to create the scores. As a result, we were able to develop an integer score-based prediction model [19].

### Internal and external validation of the model

We performed bootstrap validation 100 times as an internal validation [20]. We evaluated the internally validated model performance based on the calculated performance optimism. For external validation, we used a developed prediction model for our validation cohort (geographic validation) and evaluated the model performance of both calibration and discrimination [20]. Calibration showed the accuracy of absolute risk estimates, whereas discrimination showed how well the developed model differentiated those at higher risk from those at lower risk [9]. Model performance was calculated using the Hosmer–Lemeshow test, the description of the calibration slope for evaluating the calibration ability, and the descriptions of the receiver-operating characteristic (ROC) curve and the area under the curve (AUC) for evaluating discriminative ability [20]. For the secondary outcomes, we developed and validated prediction models and calculated the model performance in the same way.

### Comparison with the triple D score and assessment of test performance

We applied a triple D score [12] to our validation cohort. We calculated the sensitivity, specificity, and likelihood ratios of the developed model for all possible cut-off scores.

### Statistical analysis

Data were analysed using STATA version 15.0 (Stata Corp., College Station, TX, USA). The statistical significance of the Hosmer–Lemeshow test was set at *P* > 0.05.

### Missing values

To compensate for missing values, we applied multiple imputation by a chained equation, which created 20 multiple imputed datasets, and the estimates were created by combining results from multiple imputed datasets using Rubin’s rule [21].

## Results

### Patient characteristics

Figure S1 shows the flow diagram of this study. Table 1 shows patient characteristics. There were 1666 eligible patients in the model development cohort and 605 eligible patients in the model validation cohort. The average age was 55.1 years for the development cohort; it was 53.0 years for the validation cohort. Males comprised 75.0% of the development cohort and 81.8% of the validation cohort. The most common diagnosis was upper ureter calculi (61.8% in the development cohort and 59.0% in the validation cohort).

**Table 1:** Patient characteristics

	Development cohort (*n* = 1666)			Validation cohort (*n* = 605)		
	*n*	Summary		*n*	Summary	
Age (years; mean, SD)	1660	55.1	13.9	605	53.0	13.1
Gender (male; *n*, %)	1666	1250	75.0	605	495	81.8
Presence of colic (present; *n*, %)	1658	1429	86.2	598	471	78.8
Localization of calculi (*n*, %)	1664			603		
Upper calyx		10	0.6		7	1.2
Middle calyx		26	1.6		27	4.5
Renal pelvis		52	3.1		57	9.5
Upper ureter		1028	61.8		356	59.0
Middle ureter		154	9.3		47	7.8
Lower ureter		394	23.7		109	18.1
Maximum stone diameter (mean, SD)	1659	6.9	2.6	605	7.3	2.9
Skin-to-stone distance (mm; mean, SD)	1658	110.8	16.9	602	113.6	18.2
Mean stone density (HU; mean, SD)	1643	554.4	279.1	605	698.0	237.7

### Differences between the development cohort and validation cohort

The development cohort included patients from hospitals in western Japan (Okayama Prefecture, Hiroshima Prefecture, Ehime Prefecture). In contrast, our validation cohort included patients from a hospital in eastern Japan (Chiba Prefecture).

### Observed SWL failure

The development cohort included 182 patients with SWL failure after 3 sessions. The validation cohort included 111 patients with SWL failure after 3 sessions (Table S1).

### Development of the clinical prediction model

Results of the multivariable logistic regression analysis after multiple imputation are shown in Table 2. Table S2 shows the actual score calculation table. The developed prediction model was called the S_3_HoCKwave score; its name was based on the initials of the selected predictors (sex, SSD, size, Hounsfield units, colic, and kidney or ureter). The lowest score was 0 points, and the highest score was 49 points. Higher scores predicted a higher risk of SWL failure.

**Table 2:** Multivariable logistic regression analysis after multiple imputation

	Odds ratio	Standard error	95% confidence intervals	*β* coefficients	Score
Gender (ref. female)					
Male	1.29	0.26	0.87–1.92	0.26	3
Presence of colic (ref. present)					
Absent	1.31	0.28	0.87–1.99	0.27	3
Localization (ref. middle ureter)					
Kidney	1.85	0.78	0.81–4.22	0.62	6
Upper ureter	1.23	0.39	0.66–2.29	0.21	2
Lower ureter	2.37	0.81	1.21–4.64	0.86	9
Maximum stone length (ref. < 5 mm)					
5–10 mm	2.11	0.64	1.17–3.81	0.75	7
10 mm ≤	5.17	1.77	2.64–10.12	1.64	16
SSD (ref. < 120 mm)					
120 mm ≤	1.30	0.23	0.91–1.85	0.26	3
Mean stone density (ref. < 500 HU)					
500–1000 HU	2.95	0.62	1.96–4.44	1.08	11
1000 HU ≤	4.63	1.31	2.66–8.06	1.53	15

### Model performance

The performance of the S_3_HoCKwave score after internal validation and external validation is shown in Figure S2. The apparent statistical significance of the S_3_HoCKwave score was *P* = 0.49 according to the Hosmer–Lemeshow test, and the AUC was 0.75 (95% confidence interval [CI] 0.71–0.78). As a result of internal validation, the optimism-corrected AUC was 0.72. External validation according to the Hosmer–Lemeshow test showed statistical significance of *P* = 0.33, and the AUC was 0.71 (95% CI 0.65–0.76). The relationship between the score and the predicted probability is shown in Figure S3, and the test performance of the S_3_HoCKwave score is shown in Table S3.

### Performance of the triple D score used in our validation cohort

When the Triple D score was applied in our validation cohort, the statistical significance was *P* < 0.0001 according to the Hosmer–Lemeshow test, and the AUC was 0.68 (95% CI 0.60–0.77).

### ***S***_***3***_***HoCKwave scores for one-session SWL and two-session SWL***

S_3_HoCKwave scores for one-session SWL and for two-session SWL are shown in Table S3. The calibration slope and ROC curve after external validation are shown in Figure S4.

## Discussion

### Overview

In this study, we developed and validated a new clinical prediction model called the S_3_HoCKwave score. This prediction model has two important characteristics. First, the S_3_HoCKwave score is based on the sum score and consists of only six predictors; therefore, it is very easy for clinicians to use and understand compared to the clinical nomogram [22]. In addition, the S_3_HoCKwave score preserves the AUC at more than 0.70, which is classified as moderate accuracy [23]. These characteristics indicated that the S_3_HoCKwave score is also a good tool for SDM between clinicians and patients. Second, because of the sufficient sample size obtained from various types of hospitals in various areas of Japan, the developed model had better calibration and discrimination than the Triple D score after external validation. Due to these characteristics, we believe that the S_3_HoCKwave score is a more useful clinical prediction model than others, and that it can be a better tool for SDM when determining whether SWL is appropriate. The S_3_HoCKwave score is perhaps the first prediction model that has been strongly externally validated regarding the epidemiological status to predict SWL outcomes.

### Performance and clinical application of the prediction model

When the calculated score was 35, the predicted probability of SWL failure was almost 30%. The specificity values of the model of that cut-off value were 0.95 for the development cohort and 0.91 for the validation cohort. The positive predictive values were 0.87 for the development cohort and 0.80 for the validation cohort. This means that the S_3_HoCKwave score provides information for patients at high risk for SWL failure and has good predictive ability. Therefore, if the score is 35 points or more, then we may not recommend SWL.

### Strength of our study compared with previous studies

Many prediction models have been developed [10–14]; however, most of them were nomograms and may be difficult to use in daily clinical practice because of their complexity [22]. In contrast, our prediction model using the sum score can be easily interpreted. Furthermore, the performance of previously reported prediction models has not been validated; therefore, they have not provided reliable information. In fact, the reported AUC of the triple D score was 0.78 [12], but the AUC for our cohort was 0.68. In general, the apparent performance was often overestimated; therefore, internal validation to correct model optimism and external validation are recommended [20]. Our study performed both internal and external validation and presented better performance compared with the previously reported triple D score. Most previous studies have not performed both internal and external validation. To our knowledge, the S_3_HoCKwave score is the first prediction model that has been externally validated in an epidemiologically robust way to predict SWL outcomes accurately.

### Study limitations

This study had some limitations. First, it was a Japanese multicentre study, and only Asian patients participated. Although the most common composition of calculi is of calcium oxalate and calcium phosphate (82.8% in 2015) in Japan [2], which is comparable to those in the United States [24], the performance of our model for patients of other ethnicities is unknown. Further validation outside of Japan is necessary. Second, because the clinical prediction model used NCCT, the results are not applicable to patients who did not undergo NCCT. Although NCCT is recommended as a first-line diagnostic imaging tool because of its high sensitivity and specificity [25], a recent study recommended low-dose CT as a better diagnostic tool for urolithiasis because of its preserved diagnostic sensitivity, specificity, and reduced radiation dosage [26]. The predictors measured by NCCT (localisation of calculi, stone length, mean stone density, SSD) can be equivalently measured using low-dose CT [27]. Therefore, the S_3_HoCKwave score can be used even for patients diagnosed with upper urinary tract calculi by low-dose CT. Furthermore, we evaluated SWL outcomes using radiography, which has a lower sensitivity for small calculi than NCCT. Therefore, under diagnosis of SWL failure might exist. However, the low diagnostic sensitivity of X-ray examinations was especially observed for stone size smaller than 3 mm [28], which was smaller than our definition of clinically insignificant residual fragments. Additionally, radiographic examinations are superior to NCCT when it is necessary to limit radiation exposure [29]; therefore, we believe that outcomes measured using radiographic examinations support the usefulness of our prediction model in daily practice.

## Conclusion

We used NCCT information for SWL to develop and validate a new clinical prediction model called the S_3_HoCKwave score. This model had a higher predictive value than previous models. Furthermore, it was useful for selecting the appropriate treatment strategies and for SDM. Additional external validation and studies are needed to enable healthcare providers to use this scale in clinical settings worldwide.

## Electronic supplementary material

Below is the link to the electronic supplementary material.

Fig. S1 Flow diagram of the study patients. We obtained the data of patients with upper urinary tract calculi diagnosed by NCCT from 2006 to 2016. After exclusion, we divided the patients into two cohorts according to geographical factors. Finally, we analysed 1,666 patients in the development cohort and 605 patients in the validation cohort (TIFF 223 kb)

Fig. S2 Evaluation of the model performance. The performance of the S3HoCKwave score was preserved even in the validation cohort (TIF 1125 kb)

Fig. S3 The relationship between the score and predicted probability according to scatter plots (TIF 908 kb)

Fig. S4 Performance of the model after one-session SWL and two-session SWL after external validation. The statistical significance of the calibration performance according to the Hosmer-Lemeshow test was P = 0.39 for one-session SWL; it was P = 0.06 for two-session SWL. Discrimination values according to the AUC were 0.71 (95% CI, 0.67–0.75) for one-session SWL and 0.73 (95% CI, 0.68–0.77) for two-session SWL. Regardless of the number of sessions, the externally validated performance was almost similar (TIF 1017 kb)

Supplementary file5 (DOCX 17 kb)

Supplementary file6 (DOCX 17 kb)

Supplementary file7 (DOCX 16 kb)

Supplementary file8 (DOCX 20 kb)
