# Supplementary figures and images for: Development and validation of a prediction model for failed shockwave lithotripsy of upper urinary tract calculi using computed tomography information: the S3HoCKwave score
Source: World J Urol. 2020 Feb 22;38(12):3267–73. doi: 10.1007/s00345-020-03125-y (PMC7716893; doi:10.1007/s00345-020-03125-y)

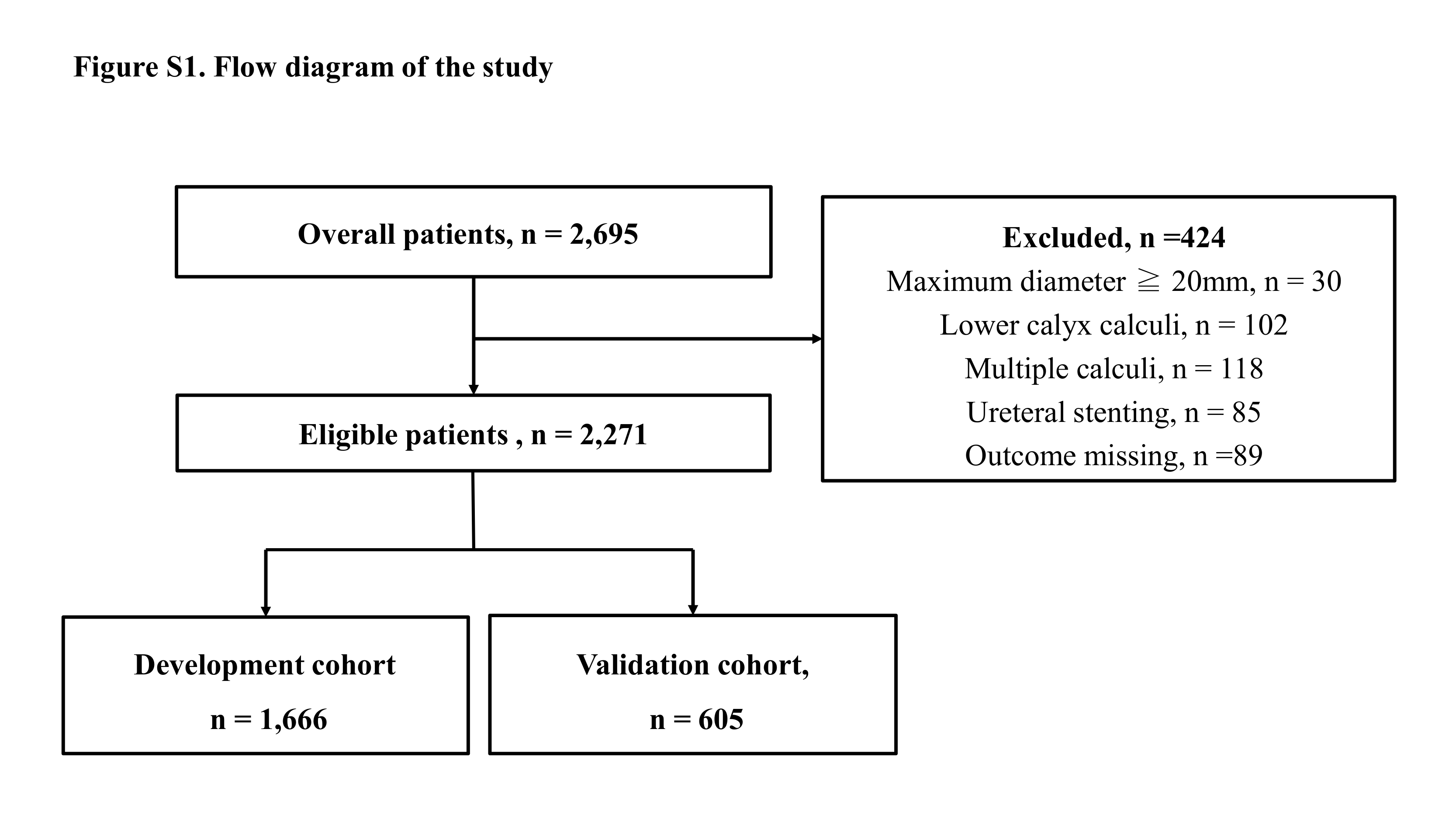

Supplement: Supplementary file 1 — Fig. S1 Flow diagram of the study patients. We obtained the data of patients with upper urinary tract calculi diagnosed by NCCT from 2006 to 2016. After exclusion, we divided the patients into two cohorts according to geographical factors. Finally, we analysed 1,666 patients in the development cohort and 605 patients in the validation cohort (TIFF 223 kb) [file 345_2020_3125_MOESM1_ESM.tiff]

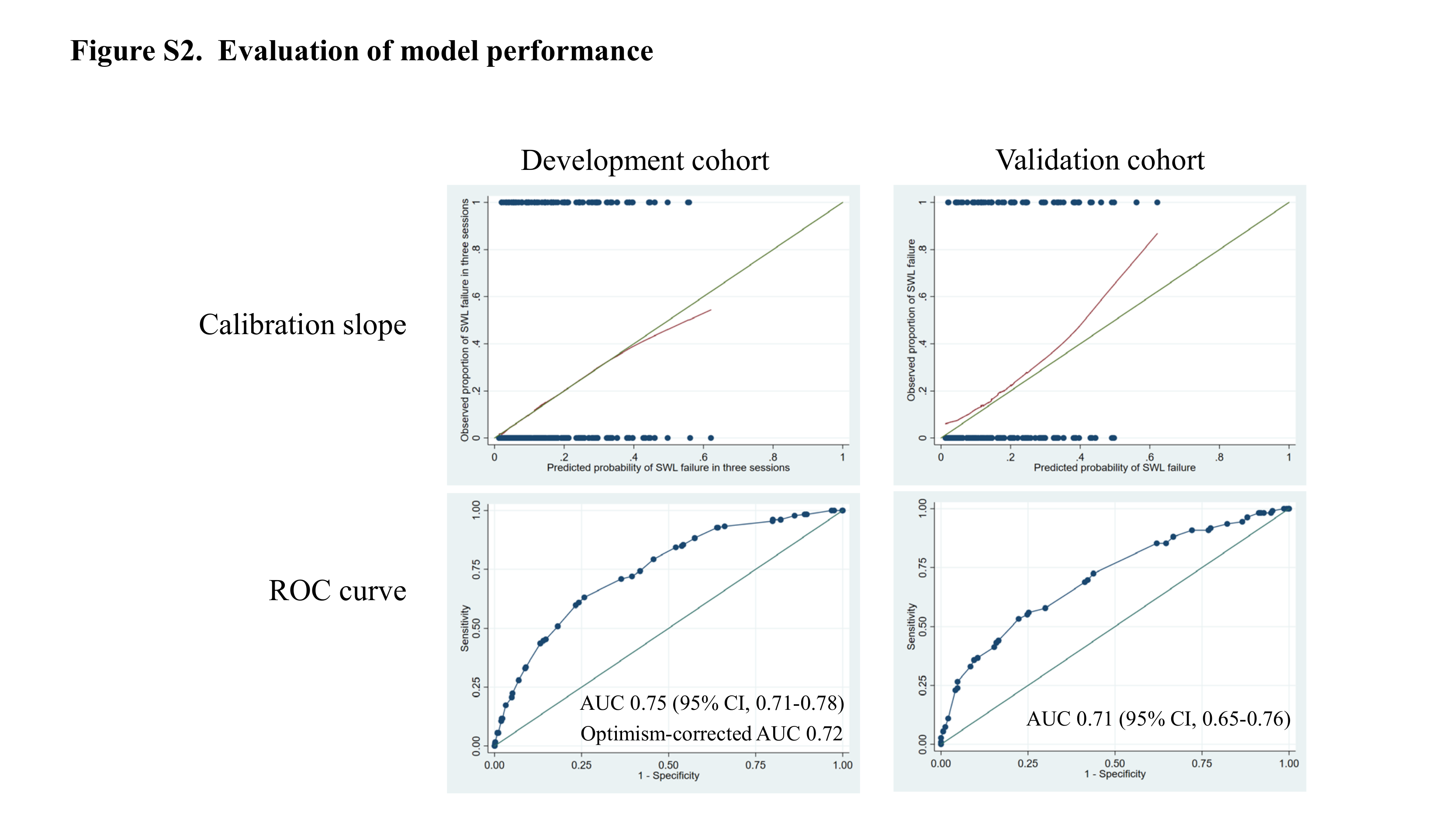

Supplement: Supplementary file 2 — Fig. S2 Evaluation of the model performance. The performance of the S3HoCKwave score was preserved even in the validation cohort (TIF 1125 kb) [file 345_2020_3125_MOESM2_ESM.tif]

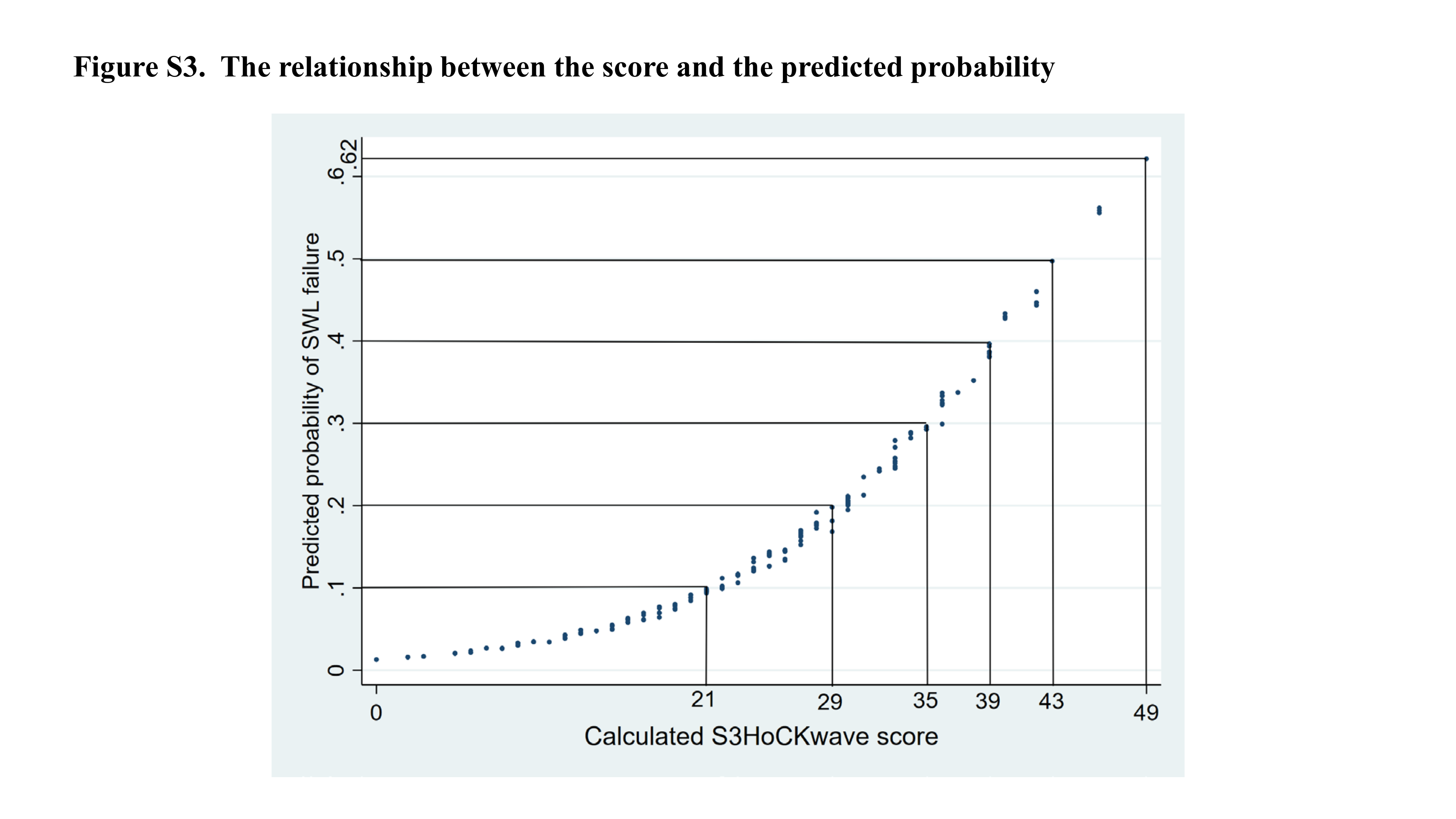

Supplement: Supplementary file 3 — Fig. S3 The relationship between the score and predicted probability according to scatter plots (TIF 908 kb) [file 345_2020_3125_MOESM3_ESM.tif]

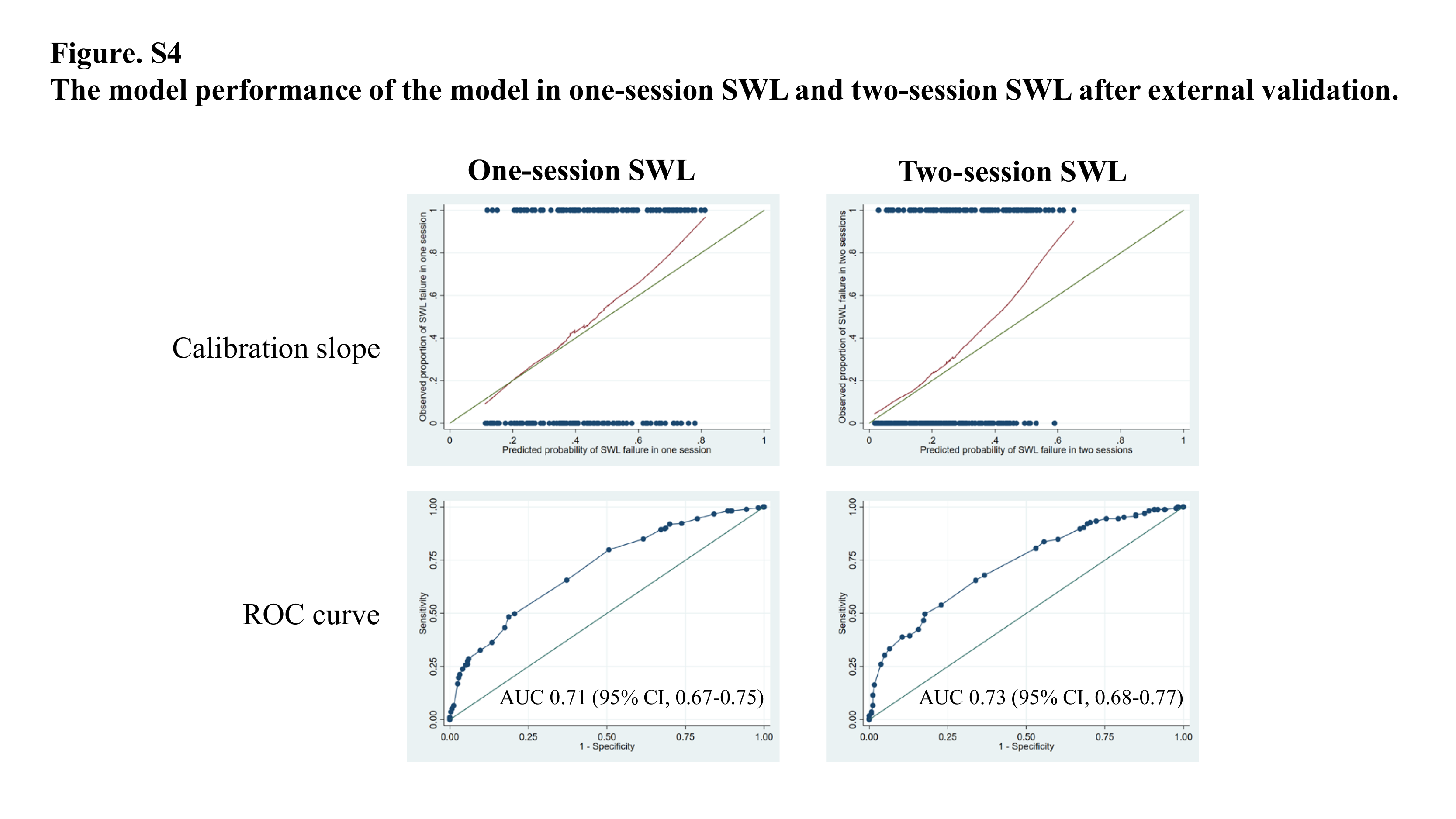

Supplement: Supplementary file 4 — Fig. S4 Performance of the model after one-session SWL and two-session SWL after external validation. The statistical significance of the calibration performance according to the Hosmer-Lemeshow test was P = 0.39 for one-session SWL; it was P = 0.06 for two-session SWL. Discrimination values according to the AUC were 0.71 (95% CI, 0.67–0.75) for one-session SWL and 0.73 (95% CI, 0.68–0.77) for two-session SWL. Regardless of the number of sessions, the externally validated performance was almost similar (TIF 1017 kb) [file 345_2020_3125_MOESM4_ESM.tif]
