# Supplementary material for: Development and validation of a prediction model for failed shockwave lithotripsy of upper urinary tract calculi using computed tomography information: the S3HoCKwave score
Source: World J Urol. 2020 Feb 22;38(12):3267–73. doi: 10.1007/s00345-020-03125-y (PMC7716893; doi:10.1007/s00345-020-03125-y)
Supplement: Supplementary file 5 — Supplementary file5 (DOCX 17 kb) [file 345_2020_3125_MOESM5_ESM.docx]

| Table S1 Characteristics of the participating hospitals and outcomes | | | | |  |  |  |  |  |  |
| --- | --- | --- | --- | --- | --- | --- | --- | --- | --- | --- |
|  |  |  |  | Development cohort, n = 1,666 | | |  | Validation cohort, n = 605 | | |
|  |  |  |  | n |  | % |  | n |  | % |
| Hospitals |  |  |  |  |  |  |  |  |  |  |
|  |  | Okayama Central Hospital |  | 1,124 |  | 67.5 |  | 0 |  | 0 |
|  |  | Kurashiki Medical Center |  | 195 |  | 11.7 |  | 0 |  | 0 |
|  |  | Onomichi Municipal Hospital | | 260 |  | 15.6 |  | 0 |  | 0 |
|  |  | Matsuyama Shimin Hospital |  | 87 |  | 5.2 |  | 0 |  | 0 |
|  |  | Abiko Toho Hospital |  | 0 |  | 0 |  | 605 |  | 100.0 |
| SWL failure in 3 sessions |  |  |  | 182 |  | 10.9 |  | 111 |  | 18.4 |
| SWL failure in 2 sessions |  |  |  | 287 |  | 17.2 |  | 168 |  | 27.8 |
| SWL failure in single session | |  |  | 608 |  | 36.5 |  | 278 |  | 46.0 |
