# Supplementary material for: Development and validation of a prediction model for failed shockwave lithotripsy of upper urinary tract calculi using computed tomography information: the S3HoCKwave score
Source: World J Urol. 2020 Feb 22;38(12):3267–73. doi: 10.1007/s00345-020-03125-y (PMC7716893; doi:10.1007/s00345-020-03125-y)
Supplement: Supplementary file 6 — Supplementary file6 (DOCX 17 kb) [file 345_2020_3125_MOESM6_ESM.docx]

| Table S2 Test performance of the SH_3_HoCKwave score | | | | | |  |  |  |  |  |  |  |
| --- | --- | --- | --- | --- | --- | --- | --- | --- | --- | --- | --- | --- |
| Development cohort | | | | | |  | Validation cohort | | | | | |
| Cutpoint | Sensitivity | Specificity | PPV | LR+ | LR- |  | Cutpoint | Sensitivity | Specificity | PPV | LR+ | LR- |
| ≥ 5 | 1.00 | 0.03 | 0.14 | 1.03 | 0.00 |  | ≥ 5 | 1.00 | 0.01 | 0.20 | 1.01 | 0.00 |
| ≥ 10 | 0.96 | 0.18 | 0.26 | 1.17 | 0.22 |  | ≥ 10 | 0.98 | 0.08 | 0.25 | 1.07 | 0.23 |
| ≥ 15 | 0.93 | 0.36 | 0.42 | 1.45 | 0.20 |  | ≥ 15 | 0.95 | 0.13 | 0.28 | 1.09 | 0.41 |
| ≥ 20 | 0.79 | 0.54 | 0.57 | 1.74 | 0.38 |  | ≥ 20 | 0.91 | 0.28 | 0.39 | 1.26 | 0.33 |
| ≥ 25 | 0.61 | 0.76 | 0.74 | 2.52 | 0.52 |  | ≥ 25 | 0.70 | 0.58 | 0.60 | 1.65 | 0.52 |
| ≥ 30 | 0.44 | 0.87 | 0.82 | 3.32 | 0.65 |  | ≥ 30 | 0.53 | 0.78 | 0.73 | 2.38 | 0.60 |
| ≥ 35 | 0.21 | 0.95 | 0.87 | 4.17 | 0.83 |  | ≥ 35 | 0.36 | 0.91 | 0.80 | 3.76 | 0.71 |
| ≥ 40 | 0.06 | 0.99 | 0.89 | 5.08 | 0.95 |  | ≥ 40 | 0.11 | 0.98 | 0.82 | 5.33 | 0.91 |
| PPV; positive predictive value, LR+; positive likelihood ratio, LR-; negative likelihood ratio | | | | | | | | | |  |  |  |
