# Supplementary material for: Development and validation of a prediction model for failed shockwave lithotripsy of upper urinary tract calculi using computed tomography information: the S3HoCKwave score
Source: World J Urol. 2020 Feb 22;38(12):3267–73. doi: 10.1007/s00345-020-03125-y (PMC7716893; doi:10.1007/s00345-020-03125-y)
Supplement: Supplementary file 7 — Supplementary file7 (DOCX 16 kb) [file 345_2020_3125_MOESM7_ESM.docx]

| Table S3 The developed "S_3_HoCKwave score". | | |  |  |  |
| --- | --- | --- | --- | --- | --- |
| Initials | Predictor | | Score | | |
|  |  |  | 3 session | 2 session | single session |
| **S** | **S**ex | Male | 3 | 3 | 2 |
| **S** | **S**SD | 120 mm ≤ | 3 | 2 | 1 |
| **S** | **S**ize | 5 -10 mm | 7 | 11 | 8 |
|  |  | 10 mm ≤ | 16 | 19 | 18 |
| **Ho** | **Ho**unsfield units | 500 - 1000 HU | 11 | 10 | 6 |
|  |  | 1000 HU ≤ | 15 | 13 | 9 |
| **C** | **C**olic | Absent | 3 | 1 | 0 |
| **K** | **K**idney or ureter | Kidney | 6 | 5 | 4 |
|  |  | Upper ureter | 2 | 3 | 1 |
|  |  | Lower ureter | 9 | 9 | 6 |
