# Supplementary material for: Development and validation of a prediction model for failed shockwave lithotripsy of upper urinary tract calculi using computed tomography information: the S3HoCKwave score
Source: World J Urol. 2020 Feb 22;38(12):3267–73. doi: 10.1007/s00345-020-03125-y (PMC7716893; doi:10.1007/s00345-020-03125-y)
Supplement: Supplementary file 5 [file 345_2020_3125_MOESM8_ESM.docx]

	Development cohort (n = 1,666)												Validation cohort (n = 605)		
	Okayama Central Hospital (n = 1,124)			Kurashiki Medical Center (n = 87)			Onomichi Municipal Hospital (n = 260)			Matsuyama Shimin Hospital (n = 195)			Abiko Toho Hospital (n = 605)		
	n	Summary		n	Summary		n	Summary		n	Summary		n	Summary	
Age (years; mean, SD)	1,120	54.6	13.8	87	55.7	14.2	259	55.2	14.6	194	57.4	13.4	605	53.0	13.1
Gender (male; n, %)	1,124	842	74.9	87	70	80.5	260	201	77.3	195	137	70.3	605	495	81.8
Presence of colic (present; n, %)	1,118	1,012	90.5	87	72	84.7	260	210	80.8	195	135	69.2	598	471	78.8
Localization of calculi (n, %)	1,123			87			259			195			603		
Upper calyx		2	0.2		0	0.0		5	1.9		3	1.5		7	1.2
Middle calyx		18	1.6		3	3.4		2	0.8		3	1.5		27	4.5
Renal pelvis		27	2.4		0	0.0		7	2.7		18	9.2		57	9.5
Upper ureter		680	60.5		66	75.9		176	68.0		106	54.4		356	59.0
Middle ureter		109	9.7		5	7.8		29	11.2		11	5.6		47	7.8
Lower ureter		287	25.6		13	14.9		40	15.4		54	27.7		109	18.1
Maximum stone diameter (mean, SD)	1,117	6.8	2.6	87	7.3	2.2	260	6.4	2.5	195	7.9	3.0	605	7.3	2.9
Skin-to-stone distance (mm; mean, SD)	1,116	111.1	16.7	87	107.6	19.0	260	112.8	16.9	195	108.0	16.4	602	113.6	18.2
Mean stone density (HU; mean, SD)	1,104	467.6	219.1	87	597.9	202.6	259	892.2	313.0	193	578.2	204.0	605	698.0	237.7
